# Successful treatment with anti-programmed-death-1 antibody in a relapsed natural killer/T-cell lymphoma patient with multi-line resistance: a case report

**DOI:** 10.1186/s12885-017-3501-4

**Published:** 2017-07-28

**Authors:** Jianping Lai, Peng Xu, Xiaoliu Jiang, Shan Zhou, Anwen Liu

**Affiliations:** Department of oncology, The Second Affiliated Hospital of Nanchang University, Nanchang, 330006 China

**Keywords:** Salvage treatment, Anti-PD-1 Antibody, Relapsed NKTCL

## Abstract

**Background:**

Extranodal natural killer/T-cell lymphoma (NKTCL), nasal type, is an aggressive malignancy with poor prognosis. Currently, there is no recommended standard therapy for relapsed NKTCL.

**Case presentation:**

A 37-year-old woman with lymphadenopathy was diagnosed with NKTCL by biopsy of an enlarged lymph node on the right side of her neck. Enhanced computed tomography revealed no metastasis. For this patient, we performed continuous chemotherapy followed by radiotherapy; however, nodule biopsy showed metastases in her lower limbs 3 months after radiotherapy, which confirmed disease progression. Unfortunately, the patient^’^ s temperature was persistently high and her skin ulcers could not be controlled well using multi-line treatment. Therefore, we attempted treatment with the anti-programmed-death-1 (PD-1) antibody, pembrolizumab. Surprisingly, the patient achieved clinical complete remission (CR) after four cycles of pembrolizumab treatment, despite having persistent detectable Epstein-Barr virus (EBV) DNA. Other molecular monitoring techniques were unavailable for this patient owing to the retrospective nature of the study. The only adverse event was soreness of the upper limb joints and muscles.

**Conclusion:**

This relapsed NKTCL case treated with pembrolizumab showed that multimodal therapy including pembrolizumab would be partially or totally effective for relapsed NKTCL.

## Background

Extranodal natural killer/T-cell lymphoma (NKTCL) is recognized as a distinct lymphoma based on the World Health Organization (WHO) classification [1]. It is found to be more prevalent in East Asia, as well as in Central and South America; simultaneously it is a rare but Epstein-Barr virus (EBV)-related lymphoma with poor outcomes [2]. Besides the nasal cavity, skin is the second most frequent extranodal site for NKTCL [3]. No randomized controlled trial has been conducted because of its rarity, and most therapeutic regimens are consensus-guided. Radiotherapy, chemotherapy, and combined chemoradiotherapy are usually effective for localized NKTCLs; however, recurrence is common. Although clinical complete remission (CR) after primary treatment has been achieved in the majority of patients [4–7], a proportion of them relapse subsequently. To date, there have been few studies on treatment of relapsed NKTCL and few results are available. The optimal management for relapsed NKTCL, especially distant recurrence, has yet to be defined.

Recently, because of the demonstration of tumor-mediated immunosuppression mechanisms, cancer immunotherapy has achieved significant breakthroughs. When immune checkpoint pathways were blocked by drugs, impressive clinical responses were observed in diverse types of human cancers [8]. Recent studies of programmed-death-1 (PD-1) blockade in lymphomas have made astounding advances, contributing to the further development of novel immunotherapies for these tumors [9]. However, the effectiveness of anti-PD-1 antibodies in patients with relapsed NKTCL is unknown. In the present case report, we describe a patient with distant relapsed NKTCL who received salvage treatment with an anti-PD-1 antibody.

## Case presentation

**Fig. 1:**
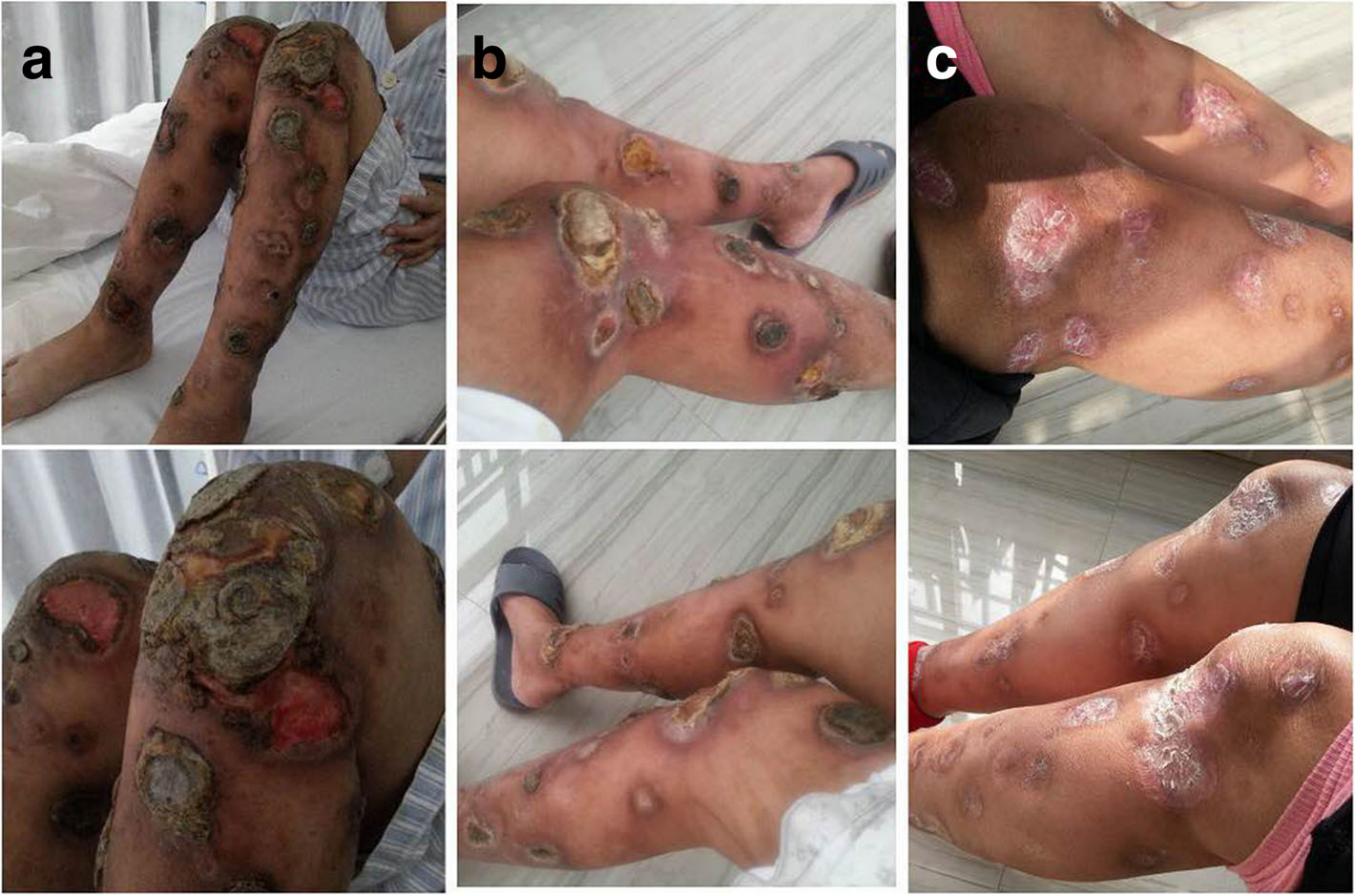
Double lower limbs ulcers after relapse. **a** Before pembrolizumab. **b** Response to pembrolizumab after one cycle of treatment. **c** Ongoing response to pembrolizumab after four cycles of treatment

**Fig. 2:**
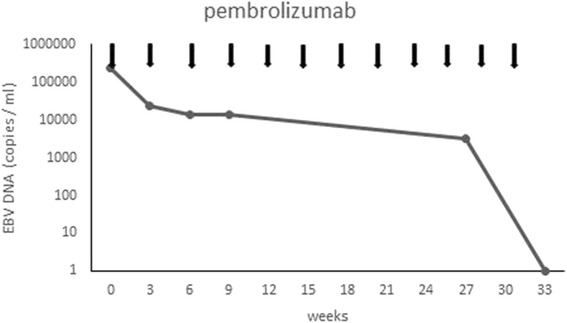
Changes in circulating EBV DNA with pembrolizumab treatment

A 37-year-old female had noticed a mass on her right neck for about 2 weeks before her initial visit to our hospital. A magnetic resonance imaging (MRI) scan of the nasopharynx and neck showed mucosal thickening in the right nasopharynx, together with multiple deep cervical lymph node enlargements. She was diagnosed with extranodal NKTCL by excisions biopsy (nasopharyngeal mass biopsy and cervical mass biopsy) and was transferred to our hospital in October 2014. Immunohistochemical staining demonstrated that the tumor cells expressed surface CD2, cytoplasmic CD3Ɛ, TIA-1, and granzyme B, but not CD10, CD15, CD20, CD21, and PAX-5. Bone marrow examination showed no presence of neoplastic cells. She was confirmed as having Ann Arbor stage IIE extranodal NKTCL based on the radiological findings and laboratory tests. She underwent four cycles of interchangeable chemotherapy comprising VIPD (etoposide, ifosfamide, cisplatin, and dexamethasone) and AspaMet (pegaspargase and methotrexate), followed by involved-field radiotherapy, and achieved complete remission. In August 2015, cutaneous nodules appeared on her lower limbs, which were proved to be relapsed NKTCL by biopsy, without involvement of the marrow. Immunophenotype showed that the nodules were CD3^+^, CD20^−^, CD30^+^, CD56^+^, CD5^−^, TIA-1^+^, Granzyme B^+^, Ki-67^+^: 95%, TCRγ^−^, and EBERs^+^. The patient developed a fever, with her temperature reaching as high as 40 °C after two cycles of the AspaMet regimen. Positron emission tomography-computed tomography (PET-CT) scans revealed multiple patchy shadows on her skin and in the subcutaneous tissue of her upper limbs and lower limbs, which accumulated radioactivity. The lymph nodes of her right armpit and bilateral groin also showed radioactive accumulation. She was switched to P-GemOx (gemcitabine, oxaliplatin, and pegaspargase) and was admitted to hospital for infected lower limb ulcers after one cycle. She was switched again to a combined Chidamide and EPOCH 80% (ifosfamide, cyclophosphamide, vincristine, pirarubicin, and dexamethasone) regimen. After the first cycle, her temperature decreased to normal levels. However, the patient developed a recurrent fever and the ulcerous areas expanded when the drugs were withdrawn (Fig. 1a). Therefore, she was treated with a combined gemcitabine, pegaspargase, dexamethasone, and doxorubicin regimen, after which the ulcerous areas narrowed and her temperature dropped slightly, accompanied by grade 4 myelosuppression. Her temperature and ulcers could not be controlled well when the fourth cycle chemotherapy was carried out. She then received another combined regimen comprising camptothecin, paclitaxel, mitoxantrone, and methylprednisolone, combined with apatinib; however, she developed infliction and chest tightness during the third day of treatment. The infliction and chest tightness remained when apatinib was given alone. An enhanced computed tomography scan showed no involvement of her organs, except the skin of lower limbs; PET-CT was unavailable. She was then treated with pembrolizumab (at a dose of 2 mg/kg every 21 days) from August 17, 2016. The skin ulcers got better after the end of the first cycle (Fig. 1b). Her performance status improved and the lower limbs ulcers had almost healed after four cycles (Fig. 1c). At this time, EBV DNA remained persistently detectable. After another seven cycles of treatment, EBV DNA became undetectable (Fig. 2). Radiological findings and PET-CT images after pembrolizumab therapy were not performed because of the patient’s refusal. The patient developed soreness of the upper limb joints and muscles, as well as a mild increase in uric acid during therapy. These symptoms were controlled well by diet control, and did not reappear after further pembrolizumab therapy. No other treatment-related adverse events were observed.

## Discussion and conclusions

The recommended standard protocol for localized NKTCL has evolved greatly over the last decade. Radiotherapy combined with chemotherapy is recommended for those cases with stage I–II nasal disease [10, 11]. However, an optimal treatment modality for relapsed NKTCL remains unclear at present. The choice of salvage treatment is associated with the type of primary regimen and response duration. Currently, the preferred treatment for relapsed NKTCLs is L-asparaginase-containing regimens, such as dexamethasone, methotrexate, ifosfamide, L-asparaginase, and etoposide (SMILE) or L-asparaginase, methotrexate, and dexamethasone (Aspa Met Dex). Kwong et al. [12] defined the efficacy of the SMILE regimen in patients with relapsed NKTCL. The Aspa Met Dex regimen was evaluated in a multicenter phase 2 clinical trial [13], where CR rate was 61.1% in 19 relapsed/refractory NKTCL patients. Efficacy of L-asparaginase-based regimen is still suboptimal for relapsed/refractory NKTCL. Gemcitabine has an impressive effect on L-asparaginase refractory NKTCL. Wang et al. [14] reported promising results for the P-GemOx regimen for newly diagnosed advanced stage or relapsed extranodal NKTCL, in which the overall response rate (ORR) was 80% (28/35) and the CR rate was 51.4% (18/35). With respect to the adverse events, grade 3/4 myelosuppression was observed in 40.0% of patients, with no treatment-related deaths. Results from a multicenter phase II study of chidamide in patients with relapsed/refractory peripheral T-cell lymphoma led to approval of chidamide by the Chinese Food and Drug Administration [15]. However, the efficacy of chidamide-based combination regimens for relapsed NKTCL is uncertain. Zhou et al. [16] found that DDGP (cisplatin, dexamethasone, gemcitabine, and pegaspargase) is an active regimen for the treatment of relapsed/refractory NKTCLs, with an ORR of 88.2% (15/17). Although promising efficacy was achieved in advanced NKTCL patients treated with a combined regimen comprising pegaspargase, l-asparaginase, and gemcitabine, a part of them still experienced failure and progression.

In the past 10 years, survival of patients has been improved greatly by antibodies targeting immune checkpoints in a number of human cancers, such as melanoma, renal cancer, colon cancer, and lung cancer. NKTCL might also be targetable for therapeutic antibodies owing to 67% of NKTCL samples expressing PD-L1 [17]. Lastly, Kwong et al. [18] reported high efficacy of pembrolizumab in relapsed/refractory NKTCL that failed on L-asparaginase. There was a obvious response to skin lesions and EBV DNA level after the first cycle in a case that had biopsy-proven cutaneous relapse. Similar results were observed in our case, which only involved the skin of the lower limbs skins. EBV DNA became undetectable only after the eleventh cycle. Our patient with only cutaneous relapse has experienced longer progression-free survival (PFS) and fewer side effects compared with those patients who had visceral organ involvement. Our case was meaningful because it proved that an anti-PD-1 antibody could be effective for selected patients with resistance to multi-line treatment.

In conclusion, we encountered a relapsed NTKCL patient with resistance to multi-line treatment, who responded well to the anti-PD-1 antibody pembrolizumab. This preliminary result suggested that pembrolizumab would be partially or totally effective for relapsed NKTCL.

## Abbreviations

Aspa Met Dex: L-asparaginase, methotrexate, and dexamethasone
AspaMet: Pegaspargase and methotrexate
CR: Complete remission
DDGP: Cisplatin, dexamethasone, gemcitabine, and pegaspargase
EBV: Epstein–Barr virus
EPOCH: Ifosfamide, cyclophosphamide, vincristine, pirarubicin, and dexamethasone
NKTCL: Natural killer/T-cell lymphoma
ORR: Overall response rate
PD-1: Programmed-death-1
PET-CT: Positron emissiontomography-computed tomography
PFS: Progression-free survival
P-GemOx: Pegaspargase, gemcitabine, and oxaliplatin
SMILE: Dexamethasone, methotrexate, ifosfamide, L-asparaginase, and etoposide
VIPD: Etoposide, ifosfamide, cisplatin, and dexamethasone
